# Functional, structural, and molecular remodelling of the goldfish (*Carassius auratus)* heart under moderate hypoxia

**DOI:** 10.1007/s10695-024-01297-7

**Published:** 2024-01-10

**Authors:** Mariacristina Filice, Alfonsina Gattuso, Sandra Imbrogno, Rosa Mazza, Daniela Amelio, Alessia Caferro, Claudio Agnisola, José Manuel Icardo, Maria Carmela Cerra

**Affiliations:** 1 Department of Biology, Ecology and Earth Sciences, University of Calabria, Arcavacata di Rende, Italy; 2 Department of Biological Sciences, University of Naples Federico II, Naples, Italy; 3 Department of Anatomy and Cell Biology, University of Cantabria, Santander, Spain

**Keywords:** Cardiac hemodynamics, Ventricular structure, Hypoxia, Goldfish, Mitochondria

## Abstract

The goldfish (*Carassius* *auratus*) is known for its physiologic ability to survive even long periods of oxygen limitation (hypoxia), adapting the cardiac performance to the requirements of peripheral tissue perfusion. We here investigated the effects of short-term moderate hypoxia on the heart, focusing on ventricular adaptation, in terms of hemodynamics and structural traits. Functional evaluations revealed that animals exposed to 4 days of environmental hypoxia increased the hemodynamic performance evaluated on ex vivo cardiac preparations. This was associated with a thicker and more vascularized ventricular compact layer and a reduced luminal lacunary space. Compared to normoxic animals, ventricular cardiomyocytes of goldfish exposed to hypoxia showed an extended mitochondrial compartment and a modulation of proteins involved in mitochondria dynamics. The enhanced expression of the pro-fission markers DRP1 and OMA1, and the modulation of the short and long forms of OPA1, suggested a hypoxia-related mitochondria fission. Our data propose that under hypoxia, the goldfish heart undergoes a structural remodelling associated with a potentiated cardiac activity. The energy demand for the highly performant myocardium is supported by an increased number of mitochondria, likely occurring through fission events.

**Supplementary Information:**

The online version contains supplementary material available at 10.1007/s10695-024-01297-7.

## Introduction

Hypoxia is a condition that commonly impacts the natural fish habitat. General physiological strategies that fishes utilize to guarantee oxygen uptake for aerobic ATP production include changes in ventilation, cardiac activity, and hemoglobin–O_2_ binding, although species-specific strategies are also possible (Mandic and Regan 2018; Mandic et al. 2009; Richards 2009; Rogers et al. 2016). Among teleost fish, species of the genus cyprinid, including the goldfish (*Carassius auratus*) and the crucian carp (*Carassius carassius*), show the capacity to survive and remain active for long periods under hypoxia, even tolerating anoxia (Bickler and Buck 2007). As firstly reported by Shoubridge and Hochachka (Shoubridge and Hochachka 1980), in the goldfish, this is correlated to a strong metabolic depression, and to the capacity to escape acidosis by converting lactate to ethanol and CO_2_, excreted through the gills.

Hypoxia resistance requires an adequate heart performance to ensure a proper tissue oxygenation and the functional and metabolic integration between organs and tissues (Filice et al. 2022). This is an intriguing trait of the remarkable and well-documented morpho-functional plasticity of the fish heart, which is proposed to be evolved as the consequence of a variety of eco-physiological challenges experienced by the animal during its life (Imbrogno and Cerra 2017; Imbrogno et al. 2019b, 2018; Olson 1998). Such flexibility is exemplified by the relationship between the structural design of the heart and its performance (Filice et al. 2022; Gamperl and Farrell 2004; Imbrogno 2013).

In *C. auratus*, the heart shows the typical fish heart organization (Farrell and Jones 1992; Icardo 2012, 2017; Tota and Gattuso 1996), being formed by four chambers in series, i.e., the *sinus venosus*, the atrium, the ventricle, and the outflow tract (*bulbus cordis*) (Garofalo et al. 2012). Due to its structure, the ventricle is an important determinant of cardiac hemodynamic. In the goldfish, the ventricular wall consists of an outer *compacta* and an inner *spongiosa* connected by a layer of collagen fibers ensuring a functional synchronism during contraction (Filice et al. 2022; Garofalo et al. 2012). The *compacta* is formed by bundles of muscle tissue variously oriented and served by coronary vessels; the underlying *spongiosa* is avascular and contains a rich network of myocardial trabeculae (Garofalo et al. 2012). This organization is the structural prerequisite that allows the goldfish heart to function as a “volume pump,” able to move large end-diastolic volumes at relatively low pressures (Filice et al. 2022; Icardo et al. 2005; Tota and Gattuso 1996).

As in other teleosts, in the goldfish, the cardiac output (CO) [(i.e., the product of heart rate (HR) and stroke volume (SV)] is mostly regulated by changes in SV than in HR (Filice et al. 2022). It has been demonstrated that the exposure of the goldfish heart to acute hypoxia significantly increases SV in a time-dependent manner (Filice et al. 2020; Imbrogno et al. 2019a, 2014; Leo et al. 2019). This behavior, particularly evident in response to preload increases [i.e., hypoxia enhances the sensitivity to heterometric regulation: (Imbrogno et al. 2014)], has been proposed as a mechanism to ensure a functional and metabolic integration between organs and tissues, crucial for preventing ethanol accumulation and intoxication.

A proper heart performance also relies on adequate energy management involving, among others, active and flexible mitochondrial equipment (Li et al. 2020). Different stimuli, including hypoxia, induce changes in mitochondrial morphology and number through the two opposite processes of fusion and fission (Adebayo et al. 2021; Forte et al. 2021; Quiles and Gustafsson 2022; Zemirli et al. 2018). Fission allows a proper distribution of mitochondria, and is crucial for removing damaged organelles, while fusion, by guaranteeing the exchange of gene products and metabolites between the fusing mitochondria, enhances the overall mitochondrial respiratory function. A failure in mitochondrial dynamics is associated with energy dysfunctions and loss of cellular homeostasis (Adebayo et al. 2021). In the mammalian heart, under oxygen deprivation, as in the case of ischemia/reperfusion, mitochondria are more prone to fission, resulting in increased mitochondria fragmentation (Brady et al. 2006; Chang and Blackstone 2010). In fish, data on the influence of oxygen deprivation on the cardiac mitochondrial compartment are scarce and fragmentary [see, for example, Gerber et al. (2021)]. In hypoxia-tolerant fish exposed to low oxygen, either a reduction of the mitochondrial compartment (Galli and Richards 2014) or no changes in mitochondrial content and activity (Gerber et al. 2019) have been reported. Recently, a normal mitochondrial respiration rate was observed in the heart of goldfish exposed for 4 weeks to severe hypoxia (PO_2_ 15.7 mmHg at 13 °C) (Farhat et al. 2021). This was accompanied by a time-dependent modulation of transcripts coding for proteins controlling mitochondrial dynamics, characterized by a general prioritization of transcripts related to pro-fusion proteins and mitochondrial biogenesis (Farhat et al. 2022).

Moved by these premises, the present study was designed to analyze whether the exposure to moderate environmental hypoxia [(i.e., PO_2_ values above the animal’s critical oxygen tension (P*crit*): (Mandic and Regan 2018)] is able to trigger a plastic response of the *C. auratus* heart. To this end, a multifaceted experimental approach was used on fish exposed to environmental normoxia (PO_2_ about 150 mmHg) or hypoxia (PO_2_ about 34 mmHg) to evaluate (i) heart performance in terms of cardiac output, stroke volume, stroke work, and heart rate; (ii) ventricular architecture, and mitochondria compartment of cardiomyocytes; (iii) expression and localization of proteins involved in mitochondrial dynamics; and (iiii) mtDNA content.

## Materials and methods

### Animals

Goldfish (*C. auratus*; length = 12–16 cm; weight = 40.96 ± 2.07 g; means ± sem, *N* = 48) specimens of both sexes were provided by a local fish farm (CARMAR, Italy). Fish were housed in tanks containing filtered, aerated, and dechlorinated tap water at 21–22 °C (12 h light/dark cycle), and daily fed ad libitum with commercial food (Premium Gold, Vitakraft, Germany). Animal care and experimental procedures were in accordance with the European and Italian laws, and approved by the Institutional Animal Care and Use Committee (CESA) of the University of Naples Federico II, Naples, Italy (N. 529/2019-PR).

### Hypoxia/normoxia exposure

Goldfish were randomly subdivided into eight glass tanks (50L), and two experimental groups (i.e., normoxia and hypoxia) were organized in quadruplicates (*N* = 6 each tank). The normoxic group was maintained at a PO_2_ of about 150 mmHg; the hypoxic group was exposed to a PO_2_ of 34 ± 3 mmHg, above the critical goldfish oxygen tension [30 mmHg: (Fry and Hart 1948; Hansen and Jensen 2010)] for 4 days. PO_2_ values were obtained by bubbling air (normoxia) or N_2_ (hypoxia) into the water. In the hypoxic tanks, water PO_2_ decreased to the desired level within 5–6 h, and then maintained stable at a value of 34 ± 3 mmHg. To limit gas exchange between water and air, aquaria were filled almost to the brim and covered with a Plexiglas lid. Water flow was set to 10 mL/min; O_2_ levels were recorded several times a day during the 4 days of treatment by using an oxygen analyzer (Milwaukee, SM600, Szeged, Hungary). During the exposure period, the temperature was kept at 21–22 °C.

The exposure time was chosen on the basis of literature data investigating the physiological mechanisms of hypoxia acclimation in fishes exposed to a similar temporal range (Paajanen and Vornanen 2003; Stecyk et al. 2004), as well as on our scientific interest to create a timeline protocol that, starting from acute hypoxia (Imbrogno et al. 2014), could furnish information on the effects of different times of exposure to environmental hypoxia on the goldfish cardiac function.

At the end of the exposure period, animals were sacrificed after anesthesia with MS222 (tricainemethanesulfonate; 0.2 g L^−1^) (Sigma-Aldrich, Milan, Italy), weighed, and then ventrally opened behind the pectoral fins. Hearts were removed and processed for the specific protocols as described below.

### *Ex vivo* working heart preparations

Hearts from goldfish exposed to normoxia (*N* = 6) and hypoxia (*N* = 6) were cannulated and connected to a perfusion apparatus as previously described (Garofalo et al. 2012). Briefly, after removing the pericardium, the heart was dissected out and placed in a dish filled with saline for cannulation procedures. Two polyethylene cannulae were secured in the ventral aorta and atrium (at the junction with the *sinus venosus*), respectively. After cannulation, the heart was transferred to a perfusion chamber filled with saline and secured with two connectors for the *sinus venosus*/atrium (input) and the ventral aorta (output) cannulae. Hearts were perfused with a solution containing (in mmol L^−1^) NaCl 124.9, KCl 2.49, MgSO_4_ 0.94, NaH_2_PO_4_ 1.0, glucose 5.0, NaHCO_3_ 15.0, and CaCl_2_ 1.2. Saline in the main reservoir was maintained at a PO_2_ of ≈ 150 mmHg (normoxia), or ≈ 30 mmHg (hypoxia) to ensure a PO_2_ reaching the isolated heart of ≈ 100 mmHg (normoxia) or ≈ 25 mmHg (hypoxia) (Filice et al. 2022). Oxygen concentration was continuously monitored by an oxygen analyzer (Milwaukee, SM600, Szeged, Hungary). pH was adjusted to 7.7–7.9 and monitored throughout the experiment. Experiments were performed at 20–21 °C. Pressures were measured with a MP-20D pressure transducer (Micron Instruments, Simi Valley, CA, USA), connected to a PowerLab data acquisition system, and analyzed using Chart software (ADInstruments Basile, Comerio, Italy). Pressure values were corrected for cannula resistance. Cardiac output (CO) was collected over 1 min and weighed. Values were corrected for fluid density and expressed as volume measurements normalized for body weight (mL min^−1^ kg^−1^). Heart rate (HR, beat min^−1^) was obtained from pressure traces. Stroke volume (SV; mL kg^−1^; CO/HR); stroke work [SW; mJ g^−1^; (afterload–preload) SV/ventricle mass].

The isolated and perfused goldfish heart generated its own rhythm. The mean output pressure was set at 1.5 kPa and the filling pressure was adjusted to about 0.07 kPa (Filice et al. 2020). Cardiac variables were simultaneously measured during experiments.

Time-course experiments were performed, and cardiac parameters were measured under basal conditions every 10 min, until 90 min of perfusion.

### Ventricular morphometry

For ventricular structure and ultrastructure, the isolated heart of goldfish acclimated to both normoxia (*N* = 3) and 4-day hypoxia (*N* = 3) was blocked in diastole with an excess of KCl, and fixed in 2.5% glutaraldehyde in phosphate-buffered saline (PBS). Samples were then processed for light or transmission electron microscopy (TEM), as described below.

To obtain semithin sections and TEM samples, small cubic pieces of tissue were removed from the middle anterior ventricular wall of glutaraldehyde-fixed hearts. Pieces were dehydrated in graded acetone and propylene oxide and embedded in Araldite (Fluka, Chemie GmbH, Buchs, Switzerland). Semithin sections, cut with a LKB III ultratome, were stained with 1% Toluidine Blue and inspected for determining orientation. Ultrathin sections, cut with a Leica Ultracut UCT, were stained with uranyl acetate and lead citrate, to be examined by a Zeiss ME 10C microscope. Micrographs of the ventricular tissue were then obtained. Images were digitized by using Olympus Camedia Z200 (GmbH, Hamburg, Germany) connected with a Zeiss III photomicroscope (thick and semithin sections).

Structural and ultrastructural parameters were measured by using the open-source NIH *ImageJ* 1.53 k software. Prior to starting analyses, geometric calibration was obtained by measuring (in pixel) the length of the ruler shown on each semithin, and the TEM picture was converted into the corresponding metric dimension.

*Compacta* thickness was evaluated on at least 12 images for each animal. By using the straight-line tool, an orthogonal segment was traced from the outer *compacta* border and the boundary between *compacta* and *spongiosa*; a minimum of 10 measures were performed on each image. To better identify the boundary between *compacta* and *spongiosa*, color pictures were converted to 8 bits, and Invert LUT was applied from the Menu Image > Lookup Tables. The same transformation was used for counting the number of vessels within the *compacta*.

To evaluate the percentage of surface occupied by *lacunae* vs *trabeculae*, the *compacta* was identified by using “*Freehand selections*” and removed from the picture. Pictures only containing the *spongiosa* were thresholded to evaluate the surface occupied by *lacunae*, the surface occupied by *trabeculae*, and the surface occupied by both *lacunae* and *trabeculae*.

Mitochondrial number and surface area were measured on at least 10 TEM images, according to Lam et al. (2021). The surface occupied by mitochondria was measured by using “*Freehand selections*” to outline each mitochondrion and measure its area. Since pictures have the same surface, the total area occupied by mitochondria was expressed as a percentage of the whole picture. Another run of counting was performed to distinguish the mitochondria located within the myofibrils and those located out of the myofibrils.

### Immunofluorescence

Immunolocalization of the dynamin-related protein 1 (DRP1) and the metalloendopeptidase OMA1, proteins controlling mitochondrial dynamics, and of Hsp90, a chaperone which contributes to cardioprotection via antioxidation and preservation of mitochondrial function (Wu et al. 2012) has been performed as here following described. Hearts from normoxic (*N* = 3) and hypoxic (*N* = 3) *C. auratus* were flushed in phosphate-buffered saline (PBS) and fixed in methanol-acetone–water solution [MAW = 2:2:1; (Garofalo et al. 2012)], dehydrated in graded ethanol (90% and 100%), cleared in xylene, embedded in Paraplast (Sigma-Aldrich), and serially sectioned at 8 μm. Dewaxed sections were rinsed in Tris-buffered saline (TBS) and incubated overnight at 4 °C with rabbit monoclonal antibodies directed against the dynamin-related protein 1 (DRP1; Novus Biologicals, cat# NB110-55288), or the metalloendopeptidase OMA1 (MyBioSource, cat# MBS3210993), and goat polyclonal antibodies directed against Hsp90 (Santa Cruz Biotechnology, #sc-1055). All antibodies were diluted 1:100 in TBS. For signal detection, after washing in TBS, slides were incubated with FITC-conjugated anti-rabbit or anti-goat IgG (Sigma-Aldrich, 1:100) and mounted with a mounting medium (Vectashield, Vector Laboratories Burlingame, CA, USA). Negative controls were obtained on parallel sections by excluding the primary antibody. Sections were observed under a fluorescence microscope (Axioscope, Zeiss, Oberkochen, Germany); images were digitized by an Axiocam camera (Zeiss).

### Mitochondria isolation

Cardiac mitochondria were isolated as described by Gerber and colleagues (Gerber et al. 2020). Ventricles from goldfish exposed to normoxia (*N* = 4) or 4-day hypoxia (*N* = 4) were thoroughly minced and gently homogenized in three volumes of isolation medium (in mmol L^−1^: 230 mannitol; 75 sucrose; 20 HEPES; 1 EGTA, pH 7.4) using an ice-cooled glass homogenizer. The crude homogenate was centrifuged at 800 g for 10 min at 4 °C to remove cell debris; the resulting supernatant was centrifuged at 8000 g for 10 min at 4 °C to pellet the mitochondria. The cytosolic fraction was recovered for further analysis, while the mitochondrial pellet was washed twice by resuspension in an ice-cold isolation medium enriched with 10 mg mL^−1^ of BSA, and centrifuged at 8000 g for 10 min at 4 °C. The final pellet was weighed and resuspended in four volumes of ice-cold isolation medium. The protein content of the mitochondrial suspensions was determined by the Bradford assay, using BSA as a standard.

### Western blotting

Western blotting analysis was performed as previously described (Mazza et al. 2019) to evaluate the expression of proteins involved in mitochondrial dynamics [i.e., optic atrophy 1 (OPA1), OMA1, and DRP1], as well as of adenosine monophosphate-activated protein kinase (AMPK), a promoter of mitochondrial fission in response to stress (Toyama et al. 2016). A 20 μg of mitochondrial proteins, or a 60 μg of cytosolic fraction, was used for the analysis. For immunodetection, blot was blocked in TBS-T containing 5% non-fat dry milk and incubated overnight at 4 °C with rabbit monoclonal antibodies directed against DRP1, or optic atrophy 1 (OPA1; Novus Biologicals, cat# NB110-55290), or OMA1, or AMPKα (Cell Signaling, cat# 2532), or Phospho-AMPK (Thr172) (40H9) (Cell Signaling, cat# 2535). All antibodies were diluted 1:500 in TBS-T containing 1% non-fat dry milk. Peroxidase-linked secondary antibodies (Santa Cruz Biotechnology Inc.) were diluted 1:1000 in TBS-T containing 5% non-fat dry milk and incubated for 1 h at room temperature. Blots were repeated twice with samples loaded in a different order. Immunodetection was performed using an enhanced chemiluminescence kit (ECL PLUS, GE Healthcare). Blot images were scanned to obtain arbitrary densitometric units. Blot signals were normalized on total proteins for mitochondrial proteins, and on β-actin for the cytosolic fraction.

### Determination of mtDNA/nDNA ratio

The quantification of the mitochondrial to nuclear genome ratio (mtDNA/nDNA), a biomarker of mitochondrial abundance and dysfunction in response to oxidative stress (Malik and Czajka 2013; Quiros et al. 2017), was performed as described in Quiros et al. (2017). Briefly, 10–20 mg of the ventricle (*N* = 4 for each condition) were thoroughly minced and homogenized in 0.3 mL of lysis buffer (0.1 M NaCl, 0.01 M EDTA, 0.5% SDS, 0.02 M Tris/HCl, pH 7.4). Samples were incubated overnight at 55 °C with 0.2 mg/mL proteinase K to degrade proteins, and then at 37 °C for 30 min with RNase A (100 μg/mL) to degrade RNA. DNA was precipitated by adding ammonium acetate (7.5 M) and isopropanol (0.7 v/v) and then centrifuged at 15,000 × g for 10 min at 4 °C. The pellet was washed twice with 70% ethanol and resuspended in TE buffer.

MtDNA content was measured using quantitative real-time PCR. Primers for mitochondrial 16S rRNA and ATP8/6 genes (accession number: NC_002079.1) and nuclear Hexokinase 2 (HK2) gene (accession number: NC_039247.1) were specific for *C. auratus langsdorfii* and *C. auratus*, respectively. Primers are listed in Table 1. Real-time PCR amplification reactions were performed via SYBR™ Select Master Mix (Thermo Fisher Scientific), according to the manufacturer’s instructions on Applied Biosystems™ QuantStudio™ 5 Real-Time PCR System apparatus. Each sample was analyzed in duplicate in 20 μL of final volume containing 6 μL Dnase/Rnase free water, 1 μL forward and reverse PCR primers at 10 μM each, and 10 μL SYBR Master Mix ready-to-use.

**Table 1:** Forward and reverse primers for real-time PCR

*Primer*	*Sequence (5’-3’)*	*Gene ID*
16SrRNA Fw	GCAAAGGTAGCGCAATCACT	NC_002079.1
16SrRNA Rev	TGAGTTGCTTAACGTGGTTGA	NC_002079.1
ATP8-6 Fw	CCACAATTAAACCCAGGCCC	NC_002079.1
ATP8-6 Rev	AGGATGGGCTTGCAAATTGG	NC_002079.1
HK2 Fw	GGCACGAATACCATCCAAGG	NC_039247.1
HK2 Rev	CTTTCCCGTGCCGCATGAAT	NC_039247.1

MtDNA/nDNA ratio has been calculated by the 2^−ΔΔCt^ method.

### Statistics and calculations

Statistical analyses were performed by using GraphPad Prism software, version 7.0 (GraphPad Software Inc, San Diego, CA, USA). All values are expressed as means ± sem of absolute values; a level of significance of *p* < 0.05 was used in all tests. The normality of data was assessed using the Shapiro–Wilk test. For the analysis of the hemodynamic parameters, a comparison between time-perfusion curves (hypoxic *vs* normoxic groups) was made by two-way ANOVA followed by Bonferroni post-test; comparison within curves (time of perfusion *vs* its control) was assessed by one-way ANOVA followed by Dunnett’s multiple comparison. For morphometric parameters and densitometric analyses, significance was assessed by unpaired Student *t*-test. Statistical analysis of real-time PCR data was performed after the 2^−ΔΔCt^ transformation; significance was assessed by one-way ANOVA followed by Tukey's multiple comparisons test.

## Results

### Hemodynamic performance

**Fig. 1:**
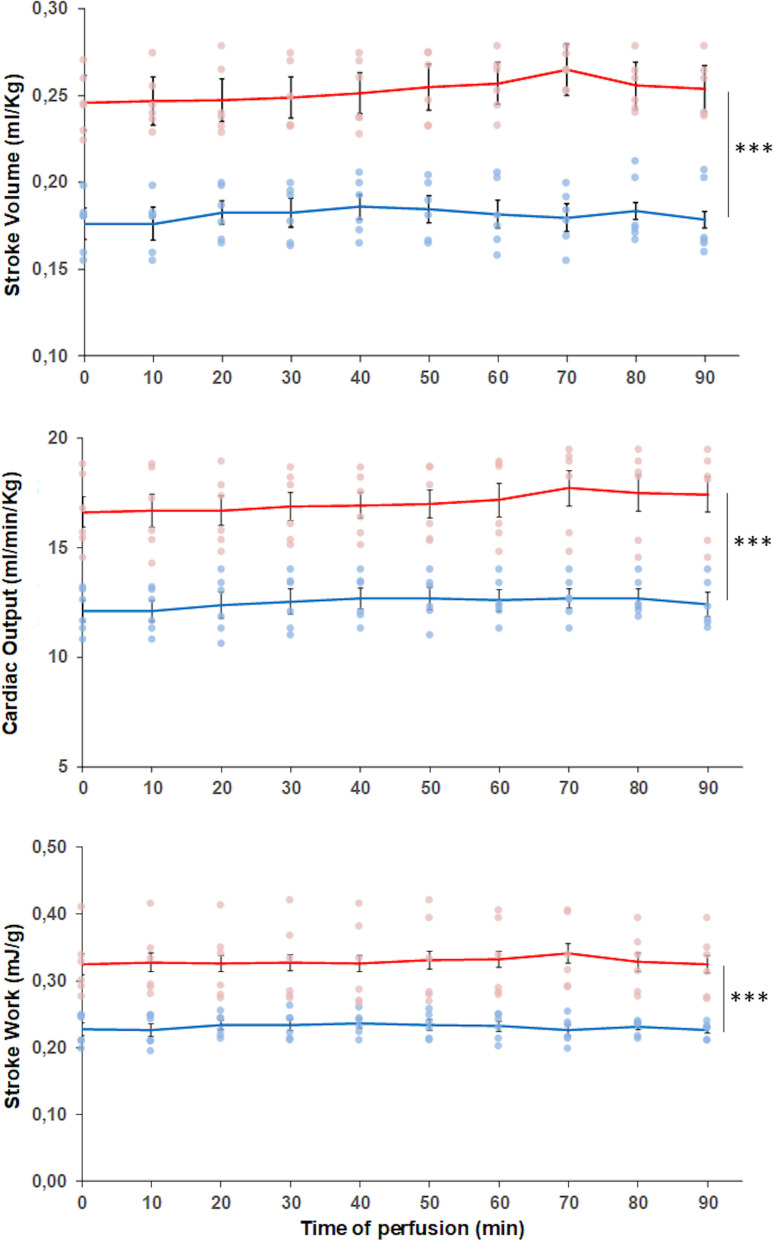
Hemodynamics of ex vivo cardiac preparations from *C. auratus* exposed to either normoxia or hypoxia. Time-course curves of cardiac output, stroke volume, and stroke work. Dots (light blue: normoxia; pink: hypoxia) represent the individual values of each experiment. Lines (blue: normoxia; red: hypoxia) represent the mean values ± sem (*N* = 6 for each condition). Statistics was assessed by two-way ANOVA followed by Bonferroni’s post hoc test (***p < 0.001; hypoxia *vs* normoxia)

The analysis of the hemodynamic parameters showed that, with respect to the normoxic counterpart, hearts from animals exposed to hypoxia exhibited significantly higher basal values of cardiac output, stroke volume, and stroke work (Table 2) which remained stable throughout the whole perfusion time (Fig. 1). No differences in basal heart rate were observed between the two experimental groups (Table 2).

**Table 2:** Basal parameters of isolated and perfused *C. auratus* cardiac preparations from animals acclimated to either normoxic (*N* = 6) or hypoxic (*N* = 6) conditions. Statistics was assessed by two-way ANOVA followed by Bonferroni’s post hoc test (***p < 0.001; hypoxia *vs* normoxia)

	Input pressure (kPa)	Mean output pressure (kPa)	Heart rate (beats/min)	Cardiac output (mL/min/kg)	Stroke volume (mL/kg)	Stroke work (mJ/g)	Power output (mW/g)
Normoxia	0.068 ± 0.005	1.395 ± 0.014	69.33 ± 3.70	12.126 ± 0.405	0.176 ± 0.007	0.228 ± 0.009	0.261 ± 0.011
Hypoxia_4d	0.067 ± 0.001	1.390 ± 0.013	67.83 ± 2.98	16.628 ± 0.694^***^	0.246 ± 0.007^***^	0.325 ± 0.019^***^	0.364 ± 0.015^***^

### Ventricular morphometry

**Fig. 2:**
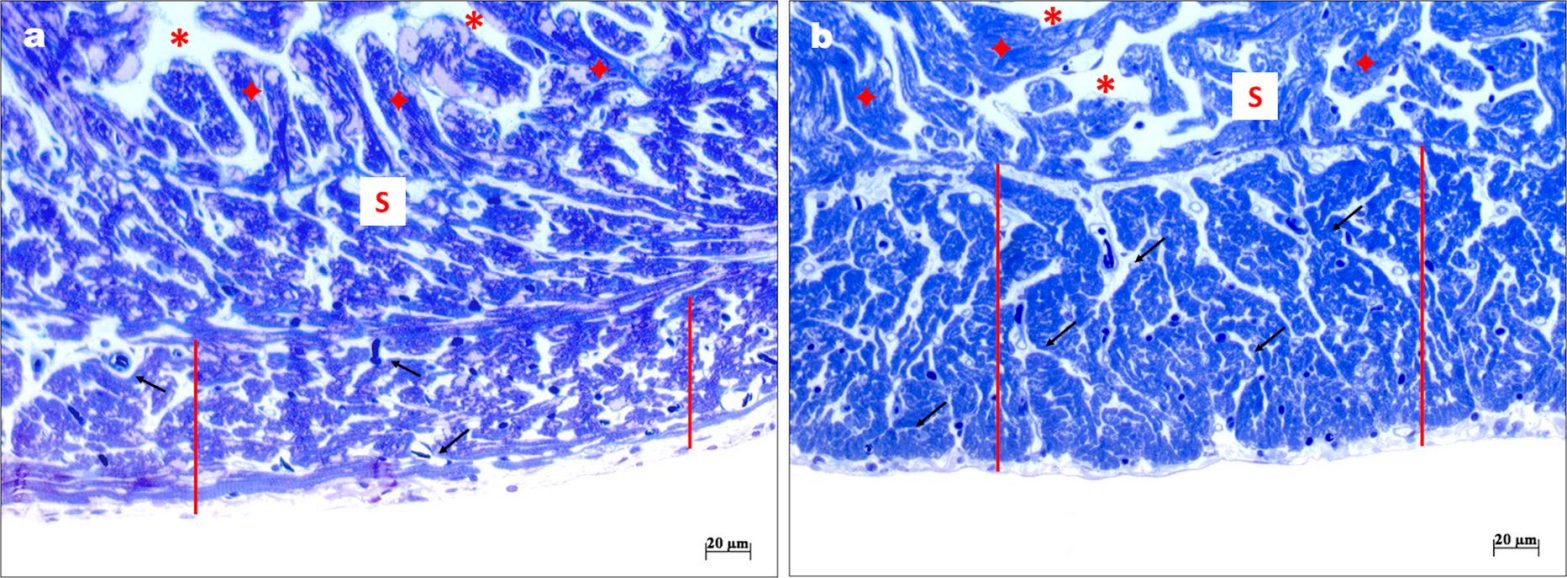
Morphological traits of the heart of *C. auratus* exposed to either normoxia or hypoxia. Representative light microscopy images of ventricular semithin sections showing the *compacta* (red line) and the *spongiosa* (S); lacunary spaces (red asterisks), trabeculae (red stars). Several vessels are indicated by black arrows. Normoxia (**a**); hypoxia (**b**)

The goldfish ventricle showed a structural organization typical of the teleost ventricle, consisting of an outer vascularized *compacta* and an inner avascular *spongiosa* (Icardo 2012, 2017). Under hypoxia, a significant increase in the *compacta* thickness and in the surface occupied by the trabeculae was observed together with a decrease in the lacunary surface (Fig. 2). These changes are paralleled by a significant increment in the number of blood vessels in the *compacta* (Table 3). No significant changes were observed in heart weight (normoxia: 0.049 ± 0.004; hypoxia: 0.046 ± 0.004).

**Table 3:** Morphometric parameters measured on semithin sections of *C. auratus* ventricle. Values were obtained on at least 12 images for each animal (*N* = 3 for each condition). *Compacta* thickness value represents the mean ± sem of a minimum of 10 measurements for each image (***p < 0.001; ****p < 0.0001)

	Normoxia	Hypoxia_4d
Ventricular structure		
* Compacta* thickness (µm)	73.10 ± 1.34	139.81 ± 2.99^****^
Trabecular surface (% of *spongiosa* surface)	63.36 ± 0.86	69.39 ± 1.1^****^
Lacunary surface (% of *spongiosa* surface)	41.06 ± 1.19	34.09 ± 1.46^***^
Vessel number (per surface units; mm^2^)	1011.38 ± 39.86	1497.42 ± 49.92^****^

**Fig. 3:**
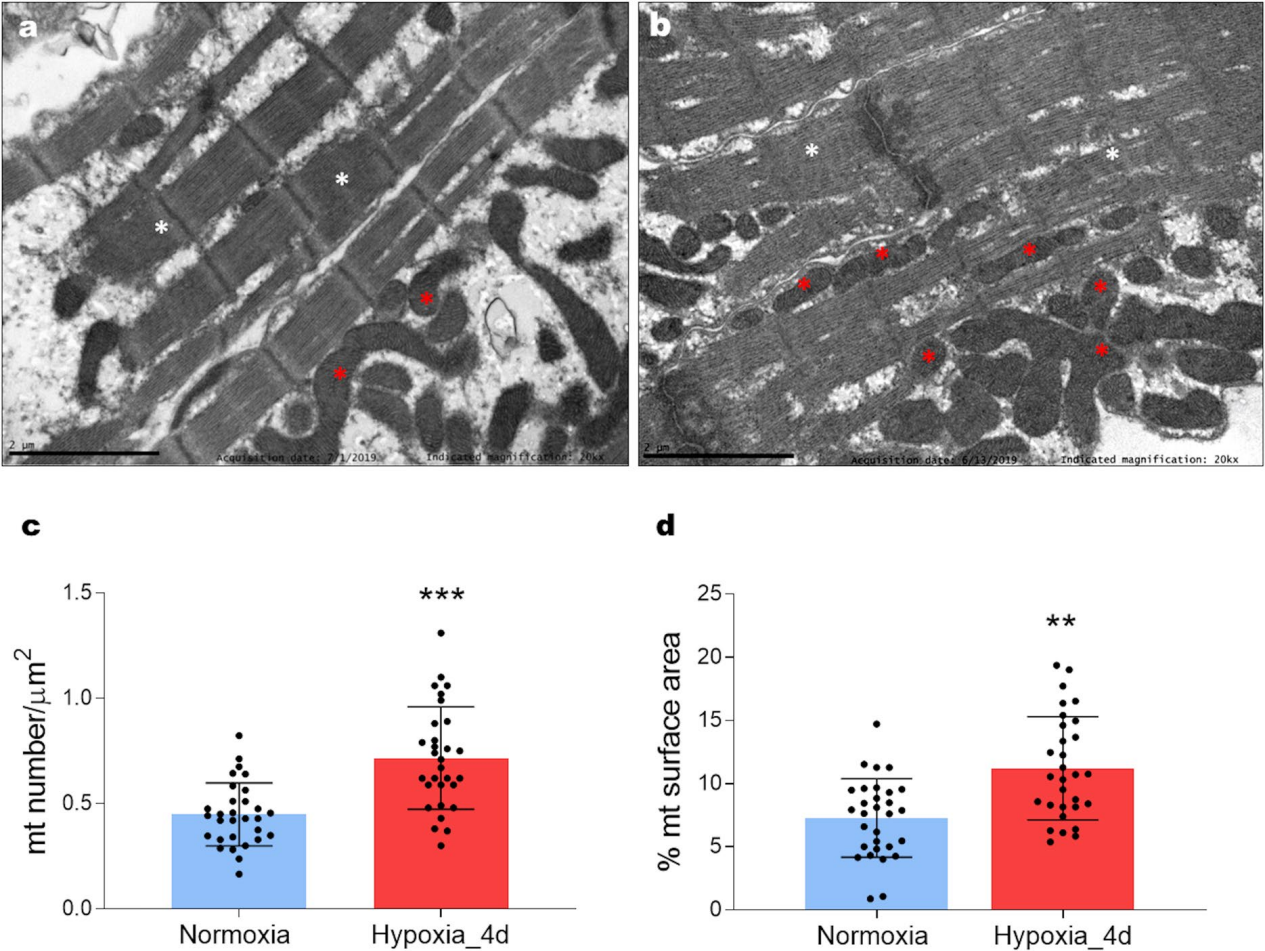
Ultrastructural traits of the heart of *C. auratus* exposed to either normoxia or hypoxia. Representative transmission electron microscopy images of the ventricle showing mitochondria (red asterisks) and myofibrils (white asterisks); normoxia (**a**), hypoxia (**b**). Bar graphs represent mitochondria number (**c**), and mitochondrial surface area (**d**). Data were obtained from 10 images for each animal (*N* = 3 for each condition). Statistics was assessed by unpaired *t*-test (***p < 0.001; **p < 0.01)

At the intracellular level, mitochondria were mainly localized around myofibrils although some of them appeared interspersed within myofibrils (mitochondria within the myofibrils: 18.79% ± 4.03 of total mitochondria; mitochondria out of the myofibrils: 81.21% ± 4.03 of total mitochondria) (Fig. 3a, b). This distribution was unaffected by hypoxia (mitochondria within the myofibrils: 20.25 ± 4.3% of total mitochondria; mitochondria out of the myofibrils: 79.74 ± 4.31% of total mitochondria). With respect to normoxia, under hypoxia, the number of mitochondria (Fig. 3c), and their surface area (Fig. 3d) calculated per picture surface significantly increased.

### Mitochondrial remodelling

**Fig. 4:**
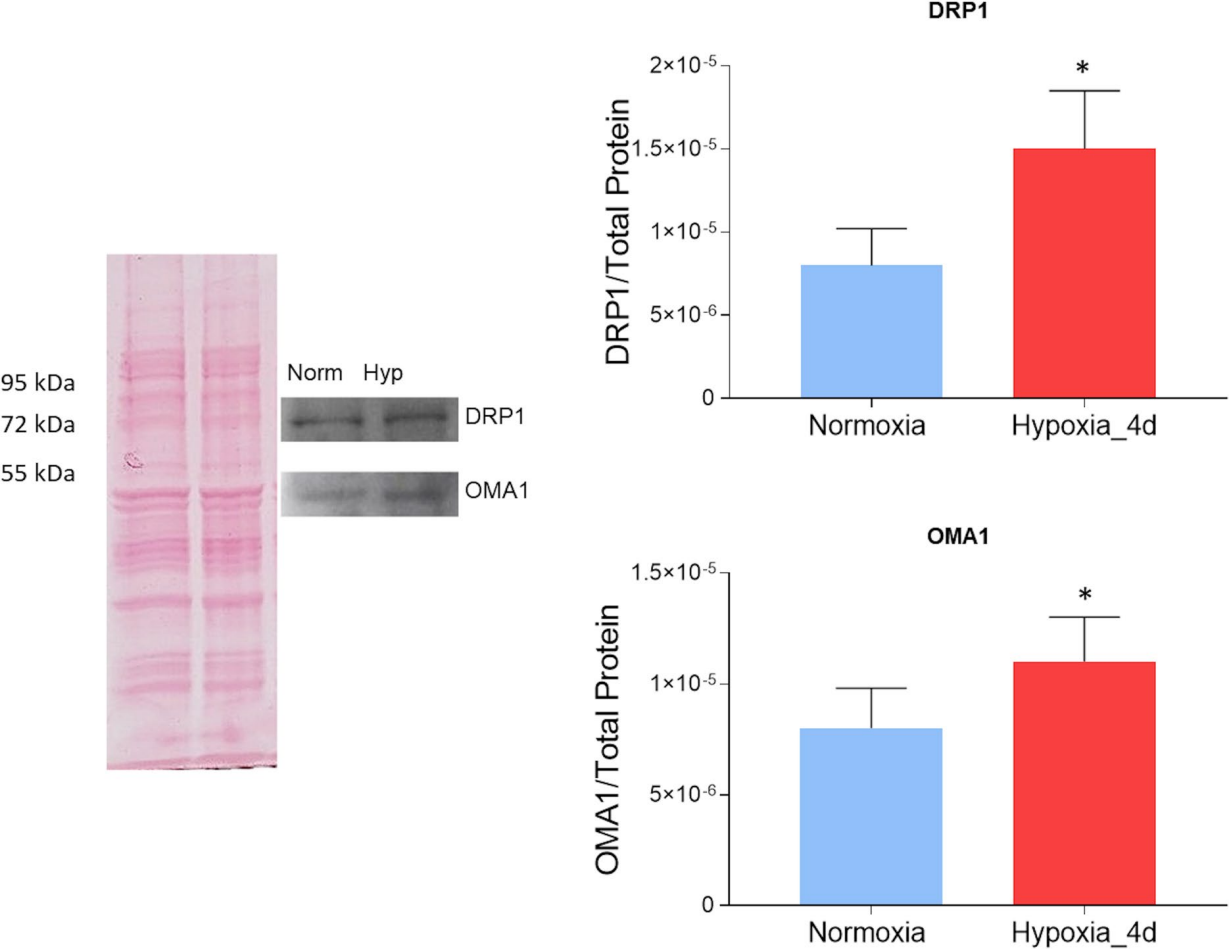
Expression of proteins regulating mitochondrial dynamics in the heart of *C. auratus* exposed to either normoxia or hypoxia. Representative Ponceau staining, blots, and densitometric analysis of DRP1 and OMA1 in cardiac mitochondria. Data were expressed as means ± sem of absolute values from individual experiments performed in duplicate (*N* = 4 for each condition). Statistics was assessed by unpaired *t-*test (*p < 0.05)

To analyze whether fission and/or fusion contribute to the mitochondrial remodelling observed in the goldfish heart under hypoxia, the expression of markers of mitochondrial dynamics was analyzed on ventricular mitochondria extracts. Western blotting and densitometric analyses showed that, with respect to the total protein content, the expression of the mitochondrial fission markers DRP1 and OMA1 increases in the mitochondrial fraction of fish exposed to 4 days of hypoxia (Fig. 4).

**Fig. 5:**
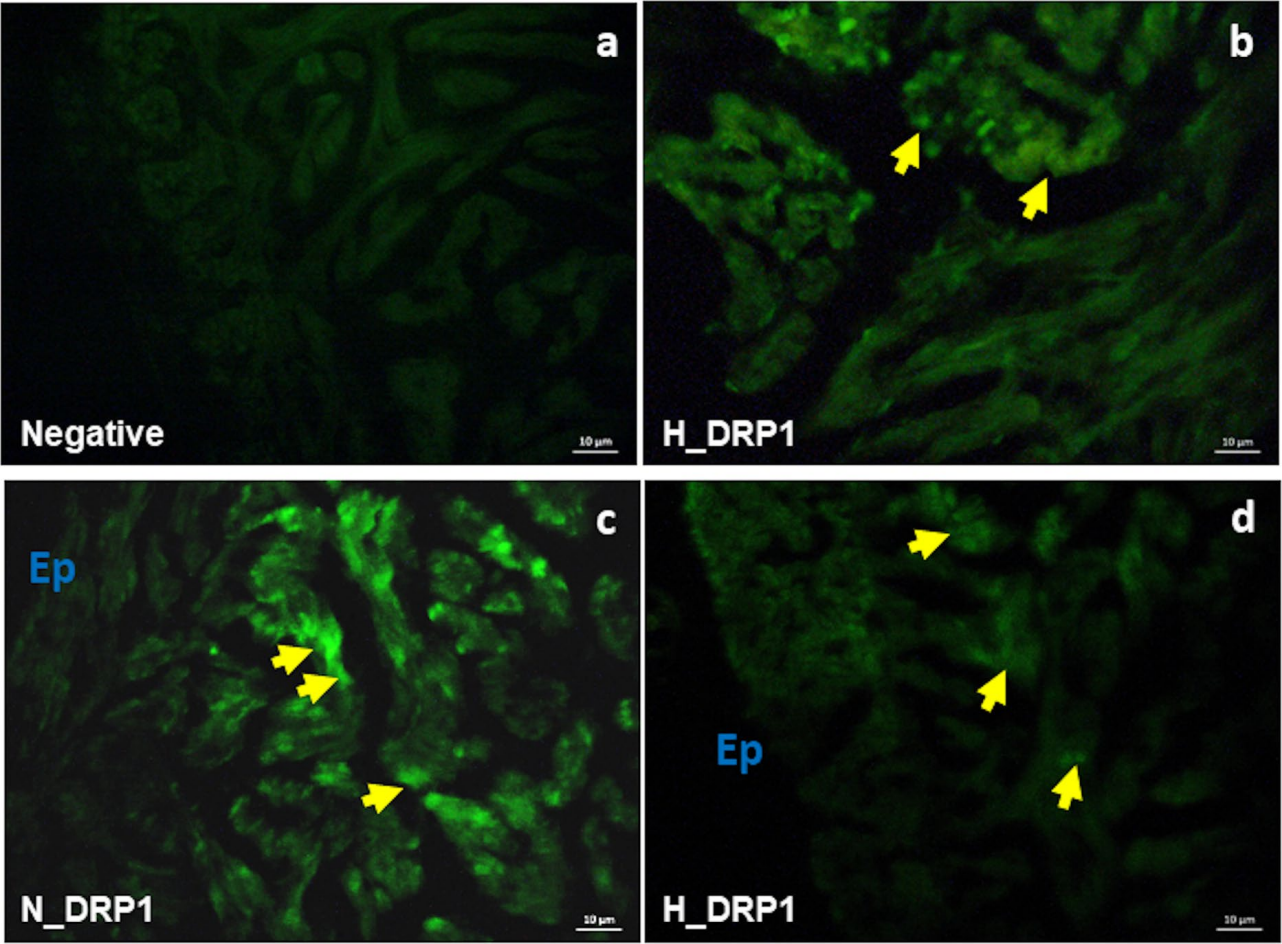
Immunolocalization of DRP1 in the heart of *C. auratus* exposed to either normoxia or hypoxia. Representative pictures showing the localization pattern of DRP1 (**b**, **c**, **d**) under normoxia (**c**) and hypoxia (**b**, **d**). Some stained cardiomyocytes (yellow arrows); epicardium (Ep). Negative control (**a**)

**Fig. 6:**
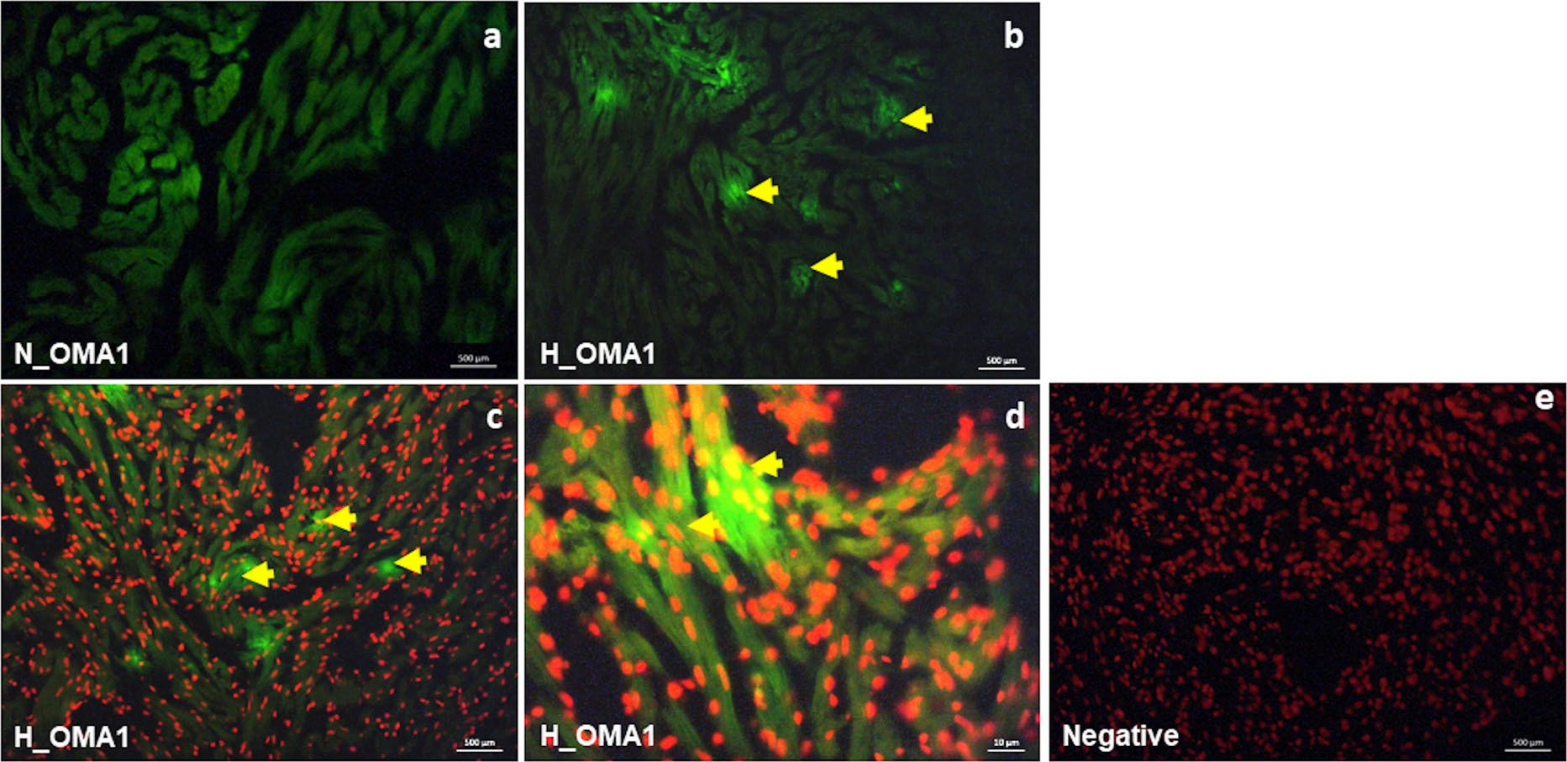
Immunolocalization of OMA1 in the heart of *C. auratus* exposed to either normoxia or hypoxia. Representative pictures showing the localization pattern of OMA1 (**a**, **b**, **c**, **d**) under normoxia (**a**) and hypoxia (**b**, **c**, **d**). Some stained cardiomyocytes (yellow arrows); negative control (**e**). In **c**, **d**, and **e**, nuclei are counter-stained with propidium iodide

Immunofluorescence observations of the ventricular distribution of the two proteins showed that in both experimental groups, DRP1 mainly localized in the cardiomyocytes of the *spongiosa* (Fig. 5b, c, d); the immunofluorescent signal appeared weak in cardiac sections from animals exposed to hypoxia (Fig. 5b, d). Contrarily, the OMA1-dependent signal was scarcely detectable under normoxia (Fig. 6a), while a randomly distributed immunofluorescence was observed in cardiomyocytes of the *spongiosa* of animals exposed to hypoxia (Fig. 6b, c, d).

**Fig. 7:**
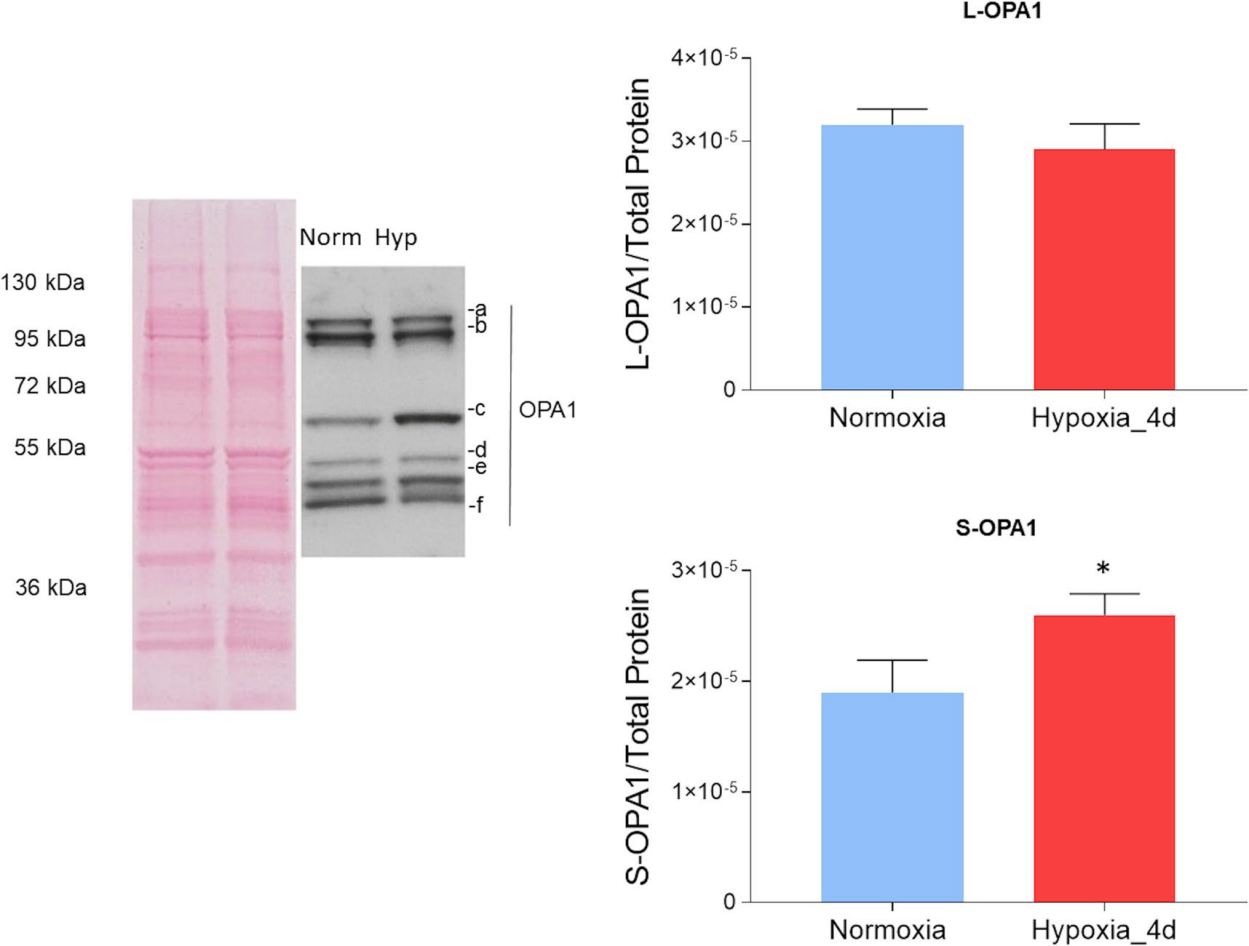
Expression of OPA1 isoforms in the heart of *C. auratus* exposed to either normoxia or hypoxia. Representative Ponceau staining, blots, and densitometric analysis of L-OPA1 (**a**, **b**) and S-OPA1 (**c**–**f**) isoforms in cardiac mitochondria. Data were expressed as means ± sem of absolute values from individual experiments performed in duplicates (*N* = 4 for each condition). Statistics was assessed by unpaired *t-*test (*p < 0.05)

Consistent with previous data in zebrafish (Bohovych et al. 2015; Rahn et al. 2013), multiple isoforms of OPA1 were detected, with a similar distribution between normoxic and hypoxic conditions (Fig. 7). Densitometric analysis of the blots revealed similar expression levels of long isoforms (L-OPA1, ~ 110–100 kDa) between the two experimental groups, while an increased expression of short isoforms (S-OPA1, between 65 and 50 kDa), indicative of an increased mitochondrial fission, was observed following hypoxia exposure (Fig. 7).

**Fig. 8:**
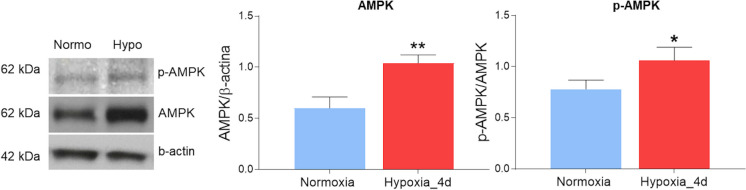
Expression of AMPK/p-AMPK in the heart of *C. auratus* exposed to either normoxia or hypoxia. Representative blots and densitometric analysis of AMPK and p-AMPK expression. Data were expressed as means ± sem of absolute values from individual experiments performed in duplicates (*N* = 4 for each condition). Statistics was assessed by unpaired *t*-test (*p < 0.05; **p < 0.01)

Western blotting and densitometric analyses of goldfish cardiac extracts showed that the adenosine monophosphate-activated protein kinase (AMPK) expression was significantly higher in animals exposed to low oxygen than in the normoxic counterpart. This was paralleled by the activation of the enzyme, suggested by the higher p-AMPK/AMPK ratio in animals exposed to low oxygen (Fig. 8).

**Fig. 9:**
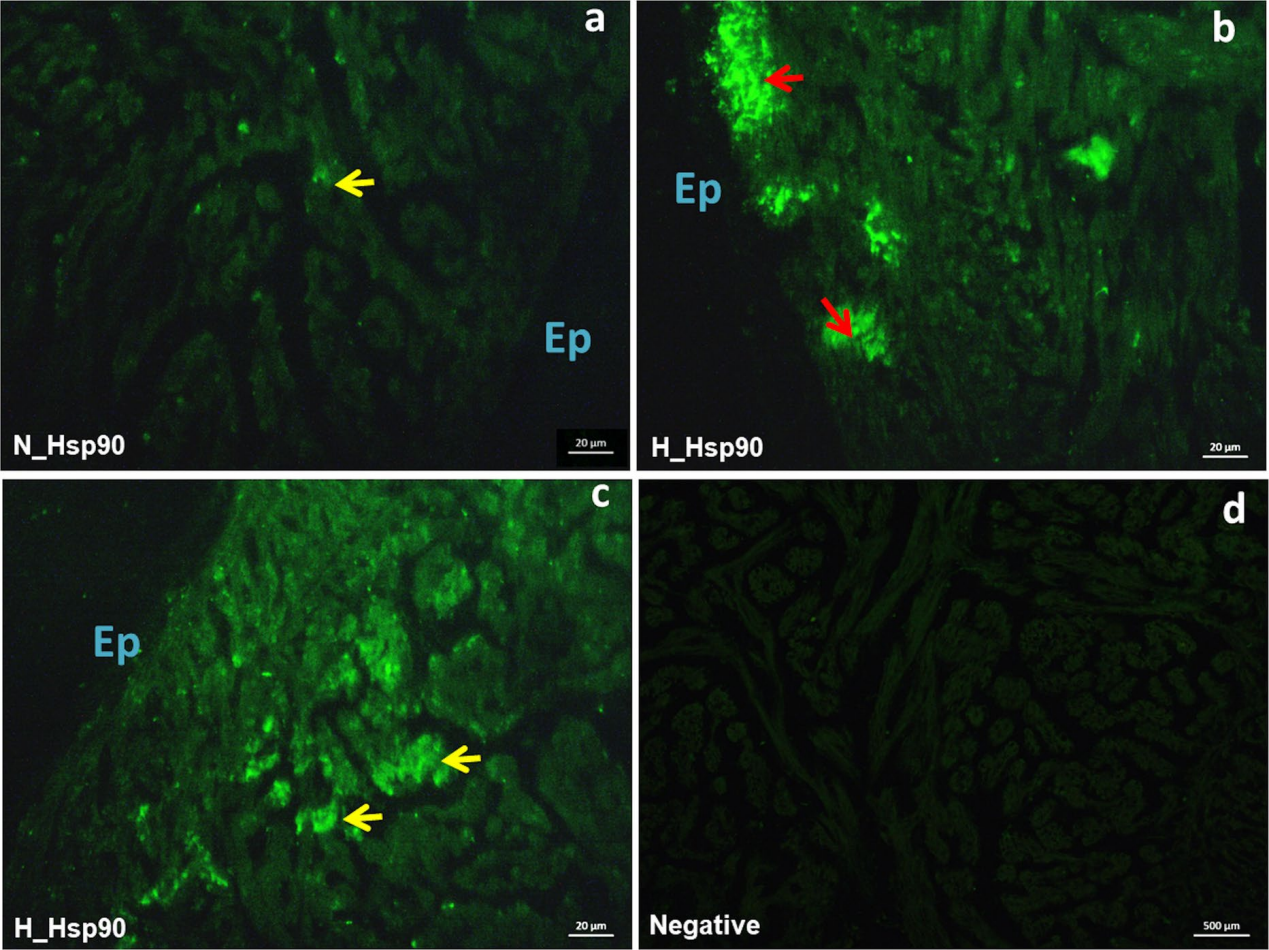
Immunolocalization of Hsp90 in the heart of *C. auratus* exposed to either normoxia or hypoxia. Representative pictures showing the localization pattern of Hsp90; normoxia (**a**), hypoxia (**b**, **c**). Negative control (**d**). Ep: epicardium, red arrows: *compacta*, yellow arrows: *spongiosa*

Immunolocalization of Hsp90 showed a weak signal mainly localized at the level of few cardiomyocytes in the *spongiosa* of normoxic hearts (Fig. 9a). Contrarily, an intense signal was observed in the heart of hypoxia-exposed fish, particularly in the *compacta* (Fig. 9b; red arrows) and in the external layer of the *spongiosa* (Fig. 9c).

### Mitochondrial DNA content

**Fig. 10:**
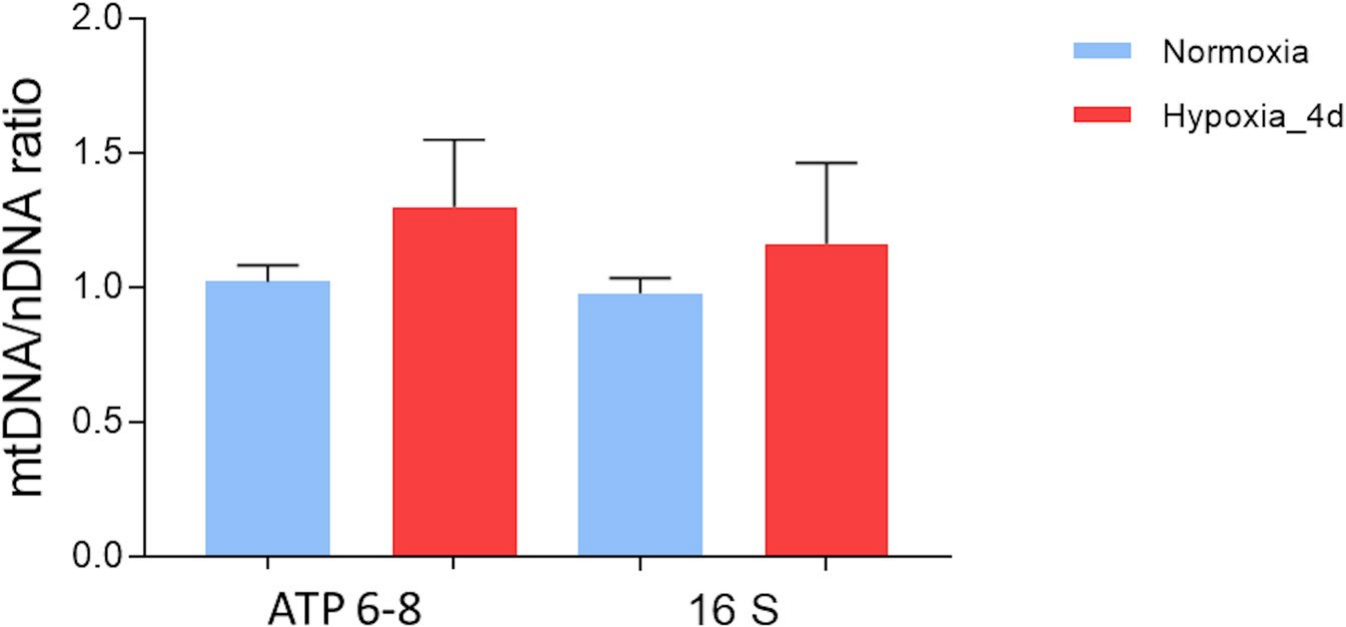
mtDNA content in the heart of *C. auratus* exposed to either normoxia or hypoxia. Relative mtDNA quantification of ATPase6/8 and 16S genes belonging to stable part of mtDNA and normalized against hexokinase 2 gene (HK2). Data were expressed as means ± sem of absolute values from individual experiments performed in duplicates (*N* = 4 for each condition). Statistics was assessed by unpaired *t*-test

The analysis of the mitochondrial DNA content, evaluated by quantitative real-time PCR as mitochondrial to nuclear genome ratio (mtDNA/nDNA), revealed no significant changes in the hearts of animals exposed to hypoxia with respect to the normoxic counterparts (Fig. 10).

## Discussion

In this study, we observed that exposure to short-term (4 days) hypoxia is associated with a functional and structural remodelling of the goldfish (*C. auratus*) heart that includes an enlargement of the ventricular trabecular myocardium, and an expansion of the cardiomyocytes mitochondrial compartment, likely occurring by fission events.

It is known that several fish species, such as the goldfish, are able to adapt their cardiac performance to tissue requirements also in the presence of low oxygen (Bickler and Buck 2007; Imbrogno et al. 2022; Stecyk et al. 2004). So far, the mechanisms that sustain this cardiac flexibility have been only partially uncovered. We here showed that ex vivo perfused working heart preparations from goldfish that had been exposed to a brief period of environmental moderate hypoxia exhibited a significantly higher cardiac performance than those exposed to normoxia. In fact, cardiac preparations of fish exposed to hypoxia showed significantly higher basal values of cardiac output, stroke volume, and stroke work, starting from the onset of the perfusion and throughout the whole perfusion time. This suggested an enhancement of the cardiac performance in animals exposed to water hypoxia. Our hypothesis is supported by the different behavior shown by the hearts of goldfish acclimated to water normoxia and acutely perfused with low oxygen. These, in fact, exhibit basal values of cardiac parameters similar to the normoxic-perfused hearts, but a gradual, time-dependent increase of SV and SW (Imbrogno et al. 2014).

In some fish, the increase of SV observed under hypoxia is related to bradycardia by a passive event linked to fiber stretching, i.e., the starling effect (Farrell 2007; Gamperl and Driedzic 2009). However, some species (e.g., *Gadhus morhua*, *Anguilla anguilla*) do not display a significant bradycardia until PO_2_ declines at levels near the *Pcrit* (Imbrogno 2013; McKenzie et al. 2009; Petersen and Gamperl 2011). Notably, goldfish exposed to water hypoxia showed basal values of heart rate that were similar to those recorded in their normoxic counterparts. This suggests that the enhanced basal hemodynamic performance observed in the isolated heart of goldfish under hypoxia occurs without significant changes in the intrinsic heart rate, mainly relying on changes in contractility. This may represent an adaptive trait that, to the best of our knowledge, has not been reported so far in the goldfish. The significance of the above changes requires deeper functional investigations. However, there may be a strategic compromise to adjust branchial performance to cope with low oxygen. In fact, increased cardiac contractility is proposed as a mechanism to enhance branchial perfusion by recruiting a larger respiratory surface under hypoxia (Booth 1979; Farrell et al. 1980, 1979; Soivio and Tuurala 1981), while bradycardia may potentiate gas lamellar transfer as a result of increased pulsatility (Daxboeck and Davie 1982). Further investigations are also necessary to determine whether, in the presence of chronic exposure to oxygen levels below *Pcrit*, the cardiac response of this teleost may be also characterized by changes in heart rate. The functional modifications observed under hypoxia were paralleled by a structural remodelling of the ventricle. This was characterized by an expansion of the trabecular myocardium accompanied by a reduction of the lacunary space, and by an increase in both *compacta* thickness and vascularization. The enhanced ventricular muscularity may call for a higher energy demand of the working myocardium, a request that can be sustained by the increased vascular supply. This possibility is supported by data in other fish species showing that the different *spongiosa/compacta* ratio and the presence of blood vessels are associated with a different hemodynamic (Cerra et al. 2004; Tota and Gattuso 1996). Under conditions requiring a potentiated performance, the compact layer and its vascularization result incremented, and this is associated with a functional shift from volume-to-pressure pump behavior (Cerra et al. 2004; Imbrogno 2013). This is the case of very active fish [e.g., tuna: (Di Maio and Block 2008)], of fish exercise trained (Farrell et al. 1990), and of those facing ontogenetic growth (Cerra et al. 2004).

As in mammals, in fish, the performance of the high energy-consuming heart relies on proper ATP availability, supported by adequate mitochondrial activity. Under various stimuli that require a rapid cardiac adaptation, mitochondria dynamically undergo changes in number, form, and intracellular distribution, thus coping with cell requirements (Sun et al. 2021). In this study, TEM analyses showed mitochondria mainly located out of the myofibrillar apparatus, but also distributed close to the sarcolemma and interspersed within myofibrils. This distribution is similar to that reported in other fish species [e.g., trout: (Birkedal et al. 2006)], but differs from the localization observed in mammals where mitochondria are orderly arranged in parallel strains within myofibrils (Birkedal et al. 2006). Although hypoxia exposure did not affect intracellular mitochondria distribution, image analyses revealed that cardiomyocytes from animals exposed to hypoxia were characterized by an increased mitochondrial surface area and number. In fish cardiomyocytes, mitochondrial remodelling represents a strategy to cope with increased heart-pumping activity. An example is the increment of the mitochondrial compartment observed in the zebrafish heart in response to hormonal stimulation (Filice et al. 2021), as well as in the ventricle of the European eel during ontogenetic growth (Cerra et al. 2004). In line with these data, our results suggest that the expansion of mitochondrial equipment observed after 4 days of hypoxia represents an adaptive mechanism that, by maximizing energy delivery to the contractile apparatus, may sustain the enhanced pumping activity of the heart.

In mammals, mitochondrial remodelling involves, among others, the regulation of fusion and fission processes (Sabouny and Shutt 2020). Under hypoxia, mammalian mitochondrial dynamics is commonly shifted toward a fission-dependent increase in number, associated with an enhanced degradation of the damaged mitochondria (Wang et al. 2022). These events contribute to mitochondria quality control when oxygen is poor or an acute energy demand occurs, and are coordinated by a balanced dynamic expression of a large set of conserved GTPases of the dynamin family (Qiu et al. 2019). DRP1 mediates mitochondrial fission, while Mitofusins 1 and 2, and OPA1 are required for the fusion of mitochondrial outer (OM) and inner membranes (IM), respectively (Adebayo et al. 2021). OPA1 activity is regulated by proteolytic cleavage mediated by OMA1, leading to the accumulation of long and short OPA1 forms. Under stress, OMA1 processes OPA1 into the short isoform, thus inhibiting fusion and triggering fission (Anand et al. 2014). Growing evidence also indicates a role of OPA1 in maintaining mitochondrial DNA (mtDNA) content, probably by anchoring this genome to the mitochondria IM (Elachouri et al. 2011).

By western blotting and immunofluorescence, we observed in the ventricle of goldfish exposed to 4 days of moderate hypoxia an increased expression of DRP1. In apparent contradiction with these data, a low DRP1 immunofluorescent signal was detected in ventricular sections of samples from hypoxic animals. Further analyses may clarify this apparent discrepancy. However, it must be underlined that DRP1 is normally present in the cytoplasm as tetramer/dimer. To promote fission, it oligomerizes upon recruitment to the outer mitochondrial membrane through receptor proteins (Smirnova et al. 2001). This may prevent the binding of the antibody to the recognition site of the protein in immunofluorescence experiments. Conversely, the denaturing conditions of the western blotting procedure may allow antibody-protein interaction, thus resulting in an evident signal.

Moreover, in hypoxia-exposed animals, we observed an increased expression of the dynamin-like GTPase OMA1, associated with a prevalent localization of the protein within cardiomyocytes. Interestingly, the OMA1 increment is accompanied by an enhanced expression of the S-OPA1 isoform, suggesting a hypoxia-related up-regulation of the fission pathways. This is supported by data showing that, under stress, OMA1 cleaves L-OPA1, causing accumulation of the pro-fission S-OPA1, thus contributing to mitochondrial fragmentation (Anand et al. 2014). As suggested in mammals (Anzell et al. 2018; Del Dotto et al. 2018; Twig et al. 2008), it is possible that fission, by segregating dysfunctional organelles, contributes to mitochondrial integrity to sustain cardiac performance under low oxygen. This is supported by evidence showing that short isoforms are more efficient in preserving mitochondrial energetics than long ones (Del Dotto et al. 2018).

Very few studies analyzed in fish the modulation of proteins involved in mitochondrial dynamics. In zebrafish, OMA1 knockout results in respiratory decline and failures in mitochondrial bioenergetics, associated with morphological cardiac defects (Bohovych et al. 2015). Also, OPA1 depletion in zebrafish embryos results in disrupted mitochondrial morphology, abnormal blood circulation, and heart abnormalities (Rahn et al. 2013). Of note, in the goldfish heart, acclimation to 1 week of severe hypoxia decreased the relative transcript abundance of the mitochondrial pro-fusion mitofusin 1 (mfn1) (Farhat et al. 2022). Together with our data, these observations suggest that under short-term hypoxia, an augmented mitochondrial number, due to the activation of fission events, may maximize energy delivery to the contractile apparatus, necessary to sustain the improved pumping behavior of the heart (Cerra et al. 2023).

To further analyze the molecular events that may contribute to the mitochondrial changes observed in the goldfish ventricle under hypoxia, we focused on AMPK, a kinase involved in a large number of cell signaling pathways, such as mitochondrial biogenesis, adaptation of antioxidant systems, and mitochondrial dynamics (Herzig and Shaw 2018; Jornayvaz and Shulman 2010). In fish, as in mammals, a wide range of stressful situations, including hypoxia, could activate AMPK (Jibb and Richards 2008; Stenslokken et al. 2008; Williams et al. 2019). In the heart of the crucian carp, 7 days of anoxia increase AMPK activation, while 10 days of severe hypoxia did not significantly affect the phosphorylation status of the protein (Stenslokken et al. 2008). In the goldfish, a very short exposure (12 h) to severe hypoxia is not accompanied by cardiac AMPK activation (Jibb and Richards 2008). In contrast, we here observed that 4 days of moderate hypoxia are associated with enhanced cardiac AMPK expression and phosphorylation. These observations suggest that in the goldfish and crucian carp, AMPK activation under low oxygen is dependent on species and time of exposure, as well as on the severity of oxygen reduction. Data in mammals also show that, under energy stress, AMPK is involved in DRP1 recruitment on the mitochondrial membrane (Ducommun et al. 2015), and this is proposed to promote mitochondrial division (Toyama et al. 2016; Wang and Youle 2016). This is interesting since it indicates a role for this kinase in the protein network controlling mitochondrial fission. Direct evidence is required to verify whether, also in the goldfish heart, the enhanced AMPK expression observed under hypoxia contributes to regulating mitochondrial fission.

Notably, the analysis of the mtDNA/nDNA ratio revealed that in the goldfish heart, even in the presence of a higher mitochondrial density, hypoxia exposure was not accompanied by significant changes in mtDNA content. This is not surprising since, while fusion contributes to maintaining mtDNA copy number through a preserved replisome protein composition (Chen et al. 2011; Silva Ramos et al. 2019), fission is important for mtDNA distribution during mitochondrial division (Lewis et al. 2016) but this does not influence mtDNA amounts (Ishihara et al. 2009).

By immunofluorescence, we also detected a strong signal of Hsp90 in the ventricle of fish exposed to 4-day hypoxia. The Hsp90 family plays a role in overseeing the protein folding environment in mitochondria. In particular, Hsp90 localizes in mitochondria and plays a role in proteostasis (Felts et al. 2000). It has been proposed that hypoxia, as well as ROS production, up-regulates the expression of these proteins and this is functionally correlated to an enhanced mitochondrial protein folding with beneficial consequences on cell survival (Altieri 2013; Wu et al. 2012).

## Conclusions

The results of this study showed that, in the goldfish, short-term exposure to moderate environmental hypoxia induces a plastic response of the heart characterized by a rapid structural and functional remodelling of the ventricle. This is associated with changes in the expression of proteins involved in mitochondria dynamics, calling for activation of the fission process. Although further studies are needed to clarify the role of mitochondrial fission in the goldfish cardiac response to hypoxia, the enhanced hemodynamics, here documented, suggest that fission is not detrimental to heart function.

The multilevel information provided by our study highlights the extreme sensitivity of the goldfish heart to changes in environmental oxygen. Our data are of relevance to better describe the physiological response to hypoxia of this cyprinid. They may also represent a useful basis to analyze whether the morpho-functional and molecular remodelling may prepare the heart to face prolonged and more severe oxygen deprivation, as experienced by the animal during its natural life. The possibility of an adaptive gradient to hypoxia of the goldfish heart is intriguing and not documented so far.

### Supplementary Information

Below is the link to the electronic supplementary material.

Supplementary file1 (DOCX 6063 KB)

## Data Availability

The datasets generated during and/or analyzed during the current study are available from the corresponding author on reasonable request.
