# Supplementary material for: Functional, structural, and molecular remodelling of the goldfish (Carassius auratus) heart under moderate hypoxia
Source: Fish Physiol Biochem. 2024 Jan 10;50(2):667–85. doi: 10.1007/s10695-024-01297-7 (PMC11021278; doi:10.1007/s10695-024-01297-7)
Supplement: Supplementary file 1 — Supplementary file1 (DOCX 6063 KB) [file 10695_2024_1297_MOESM1_ESM.docx]

N I N I N I

N I N I N I

Fig. S1. Original Blot and Ponceau staining reported in Fig. 4.

Fig. S2. Original Blot and Ponceau staining reported in Fig. 6.

N I N I N I N I

N I N I N I N I

Fig. S3. Original Blot reported in Fig. 7.


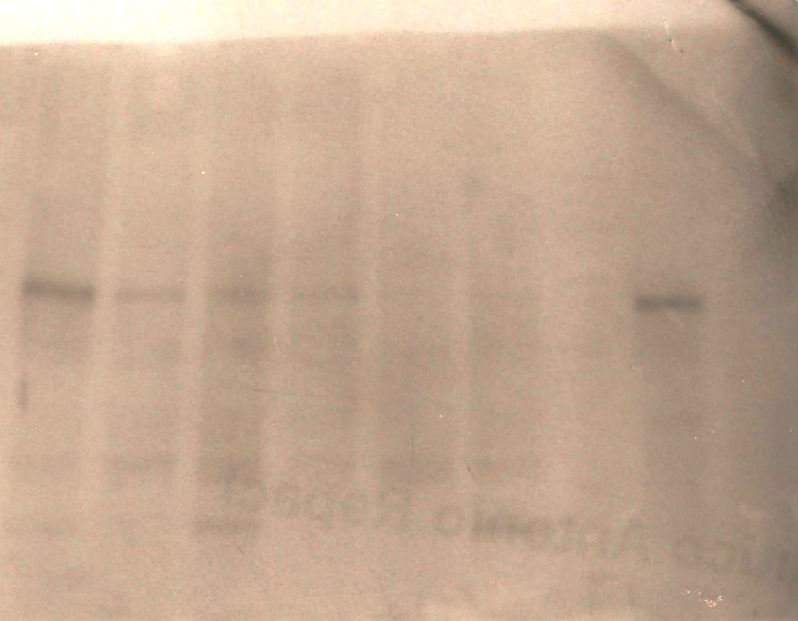


Control p-AMPK

C N I N I N I C

Fig. S4. Blot showing positive control for p-AMPK

Normoxia


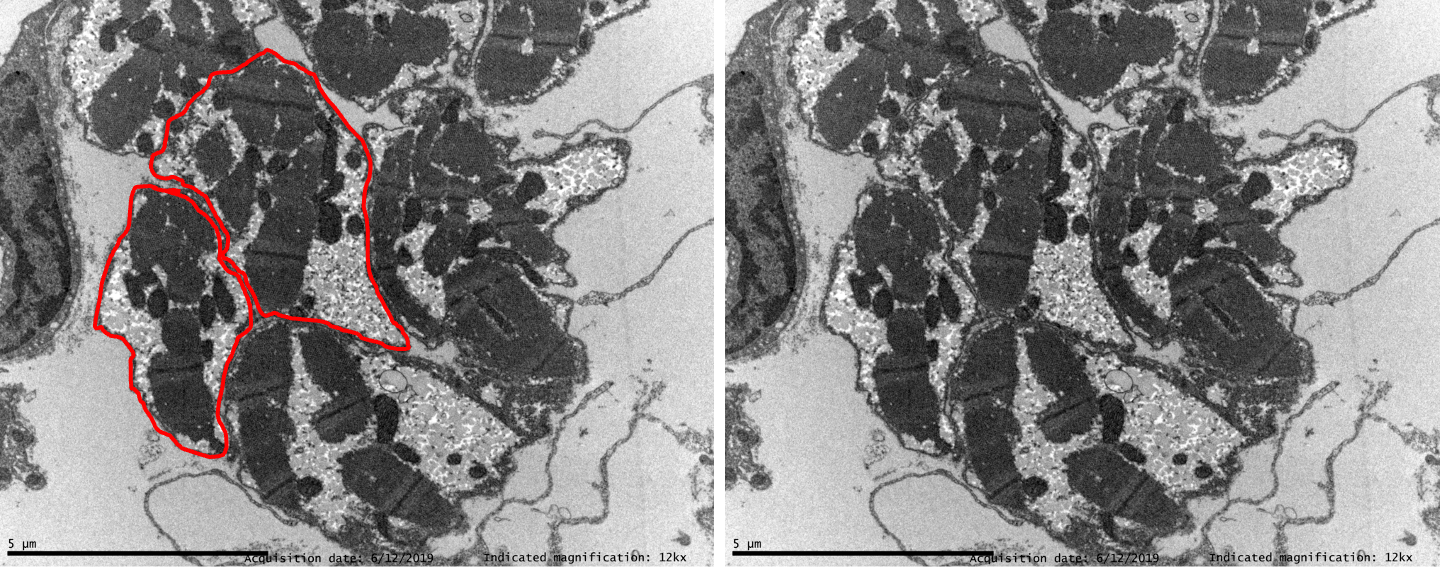


Hypoxia


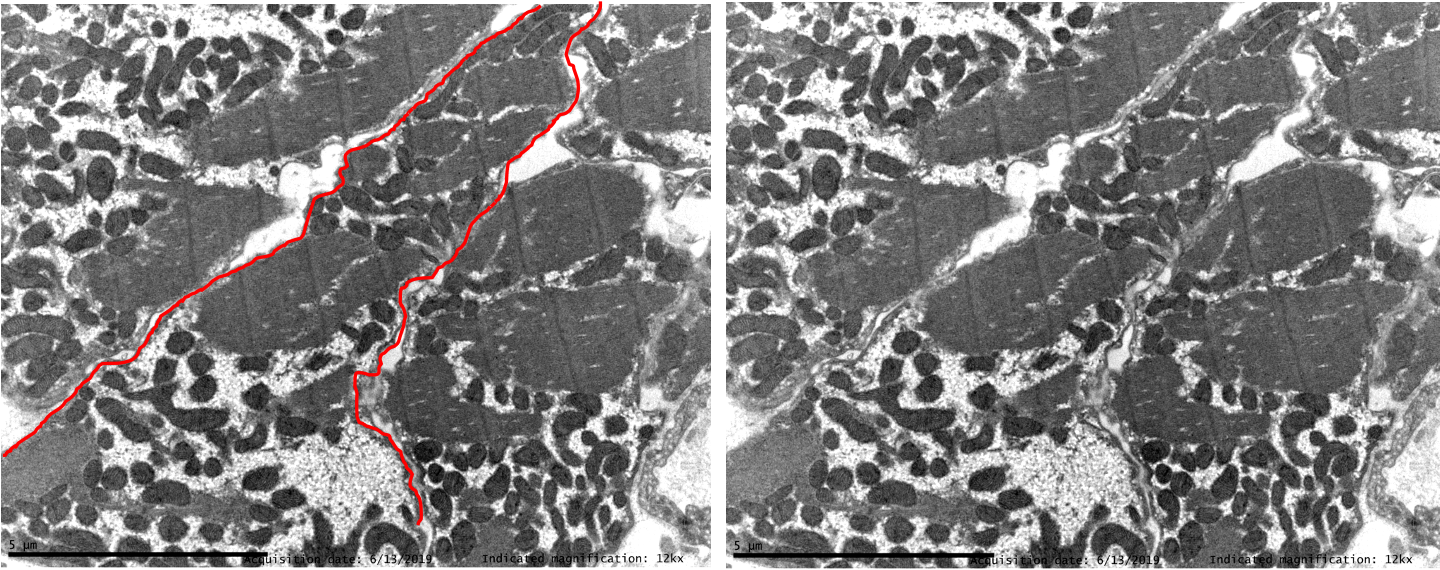


Fig.S5. Representative low magnification TEM images of the ventricular myocardium of animals exposed to both normoxia and hypoxia showing mitochondria distribution. Cells outlines are marked with a red line.
